# Single-Mode Semiconductor Nanowire Lasers With Coupled Cavities

**DOI:** 10.3389/fchem.2020.631870

**Published:** 2021-01-15

**Authors:** Salman Ullah, Sijie Pian, Fang Dai, Yilun Wang, Yaoguang Ma, Qing Yang

**Affiliations:** ^1^State Key Laboratory of Modern Optical Instrumentation, International Research Center for Advanced Photonics, College of Optical Science and Engineering, Zhejiang University, Hangzhou, China; ^2^Collaborative Innovation Center of Extreme Optics, Shanxi University, Taiyuan, China; ^3^East China Institute of Optoelectronic Integrated Device, Suzhou, China; ^4^Institute of Navigation and Control Technology, China North Industries Group Corporation, Beijing, China

**Keywords:** single-mode, semiconductor nanowire laser, nanowire laser, mode selection, coupled cavity

## Abstract

Semiconductor nanowires are one of the most fascinating topics over the past few decades. As miniaturized coherent light sources, semiconductor nanowires have been attracting tremendous attention in recent years for scientific and technological interest as potential ultra-compact, low cost, high efficiency, and low power consumption. Among different types of lasers, one-dimensional nanowires are of great interest as a promising material for next-generation nanophotonics and nanoelectronics applications due to their unique optical and electrical properties. Semiconductor nanowire lasers with single-mode output are vital in a variety of practical applications ranging from signal processing, spectroscopy, displays, optical sensing, on-chip communications, and biological studies. This article reviews the basic technology and research progress of single-mode semiconductor nanowire lasers. Afterward, the key methods and development of the different types of coupling to achieved single-mode laser output are elaborated. Finally, the challenges faced by each scheme are summarized.

## Introduction

In 1959 a lecture given by Richard Feynman at the annual meeting of the American Physical Society entitled “There's plenty of room at the bottom” addresses the challenges of “writing things small” and how to process the information on the nanometers scale (10^−9^) (Ma et al., 2013). Thereafter, in 1960 Theodore Maiman using ruby as a gain medium (Maiman, 1960) demonstrates the first working laser. Since these early developments, the use of lasers has a profound impact in various fields of materials, optics, and electronics and has become an integral part of our modern life (Eaton et al., 2016).

To accelerate the practical application of semiconductor nanowire lasers, it is necessary to control and optimize the parameters of nanolasers including wavelength tuning (Li et al., 2013; Liu et al., 2013a,b; Yang et al., 2014; Zhuge et al., 2019a,b), polarization modification (Hurtado et al., 2013; Xu et al., 2014, 2017), and mode selection (Xiao et al., 2011a,b; Li et al., 2012; Gao et al., 2013; Feng et al., 2014; Zhang et al., 2016; Yang et al., 2017; Bao et al., 2020; Yu et al., 2020). Among them, the lasing monochromaticity plays an important role as group velocity dispersion between different modes in a multimode laser will result in both temporal pulse broadening and false signaling (Saleh and Teich, 2007). Delegated mode selection methods could solve these problems by making the laser to oscillate at a single frequency. Technically, single-mode lasing can be realized when the free spectral range (FSR) is larger than the bandwidth of the optical gain (Javan et al., 1961; Zayhowski and Mooradian, 1989; Loh et al., 1998). Shortening NWs is a straightforward method so that the widening FSR can exceed the whole gain profile of materials (Binet et al., 1998; Yu et al., 1998). However, with the low reflectivity of the end facets of NWs (Maslov and Ning, 2003), such short NWs with limited gain leads to a high lasing threshold (Park and Chuang, 1998; Yamamoto et al., 1999). To address this issue, one improved approach is to use well-designed cavity structures such as cleaved-coupled nanowire cavities (Gao et al., 2013) and loop mirror structures (Xiao et al., 2011a). Fabry-Perot (FP)-Whispering gallery mode (WGM) hybrid cavity coupling based on semiconductor NWs is another effective way to efficiently introduce the Vernier effect to further broadening the FSR of the nanowire laser while increasing the Q-factor of lasing oscillator of specific wavelengths.

## Single Mode Semiconductor Nanowire Laser Based on Coupled Cavities

**Figure 1:**
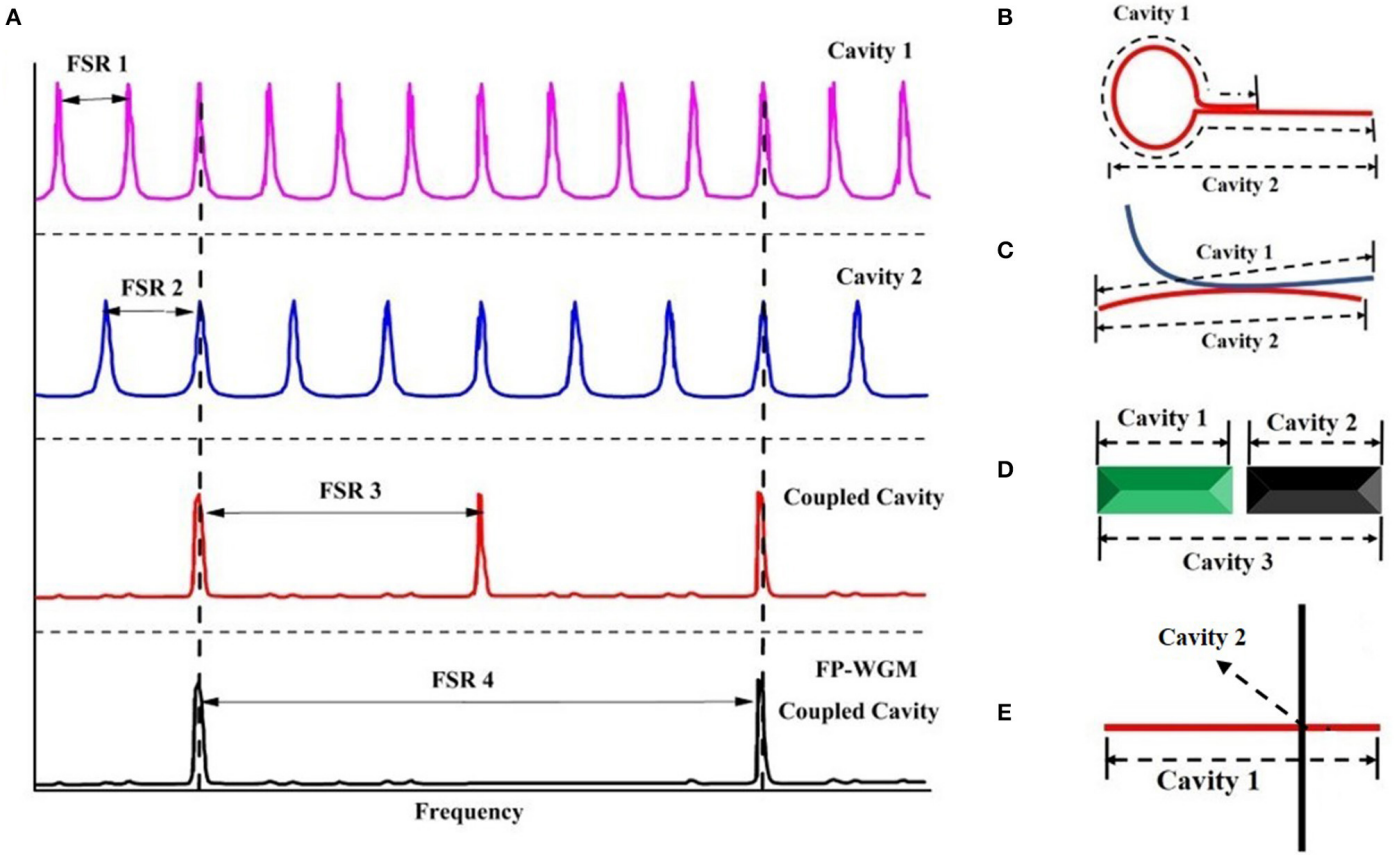
Vernier effect and schematics of typical coupled cavities nanowire lasers. **(A)** Schematic of Vernier effect; **(B)** single nanowire coupled-cavity with a loop mirror end; **(C)** X-shape cavity composed by two nanowires in contact; **(D)** cleaved coupled nanowire cavity **(E)** FP-WGM coupled nanowire cavity.

Coupled-cavities has been widely used in the traditional semiconductor lasers (Lee et al., 1985; Lang et al., 2019) fiber lasers (Fan et al., 2012), and other fields. Based on the Vernier effect (Xu et al., 2010), the resonating frequency components have to satisfy the resonance conditions of all cavities in the coupled-cavity laser. This greatly increases the FSR and Q values of the resonant cavity, making it possible to maintain a certain cavity length (which means sufficient gain length) and a single longitudinal mode output as shown in Figure 1A. According to the coupling mode between cavities, a variety of typical structures have been reported, including loop mirror, X-shaped, and cleavage coupled-cavity as shown in Figures 1B–E.

**Figure 2:**
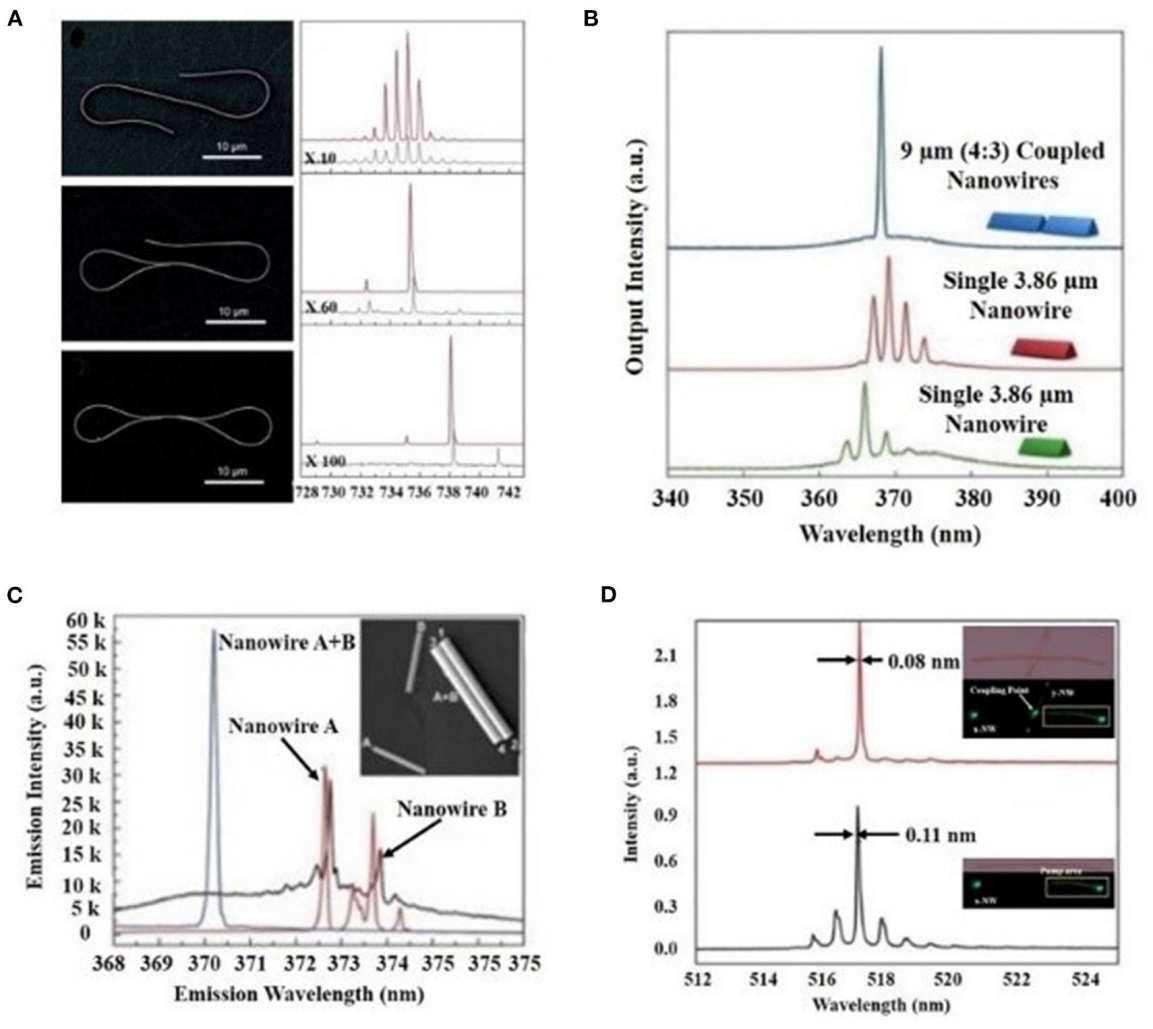
Different types of nanowire laser coupling schemes and their corresponding lasing spectra **(A)** SEM image and corresponding output spectra of the laser without a loop, with a single loop and with double loops (Xiao et al., 2011a) **(B)** lasing spectrum of cleaved-coupled nanowire laser and its spectral characteristics (Gao et al., 2013) **(C)** Lasing emission spectra of the coupled nanowire-pair and corresponding separated individual nanowires (Xu et al., 2012) **(D)** lasing spectra of single CdS nanowire laser and FP-WGM hybrid cavity coupling and their lasing spectrum.

In 2011, Xiao et al. from Zhejiang University (Xiao et al., 2011a) reported folding one or both ends of CdSe into a loop mirror through micromanipulation, and realized a single-mode laser in 700 nm wavelength range, its basic structure and output laser characteristics as shown in Figure 2A. The threshold and line width of the laser can be further reduced (threshold 34.4 μJ/cm^2^, line width 0.10 nm), and a higher side-mode suppression ratio (SMSR) can be obtained (from 9.34 to 11.3 dB). Later on, the same group reported coupling two nanowires together to form an X-shaped coupling resonator for single-mode lasing output (Xiao et al., 2011b), as shown in Figure 1C. Compared with the loop mirror structure, this kind of coupling only needs to put two nanowires together and the micromanipulation is relatively easier.

Based on a similar principle, Xu et al. (2012) achieved single-mode laser output in the UV range by close contact of two GaN nanowire with diameters/lengths of 680 nm/7.6 μm and 720 nm/8 μm, respectively, as shown in Figure 2C, and found that the Vernier effect in this structure supports an SMSR of ~15.6 dB. In 2017, Ditcovski and Ellenbogen (2017) pointed out that for two pairs of vertically coupled nanowires with the same length and different diameters, because different diameters correspond to the same equivalent refractive indexes, which results in different FSR, it still has a Vernier effect. Through numerical simulation, the research group verified the feasibility of using this method to achieve single-mode laser output. However, due to the need to precisely control the geometric parameters and relative positions of the nanowire pairs, this scheme faces certain challenges.

Based on the transverse coupling of the evanescent wave, the characteristics of the output laser (such as wavelength, threshold, coupling efficiency, etc.) are highly sensitive to the geometric characteristics of the coupling cavities (nanowire spacing, coupling length, size, etc.). Additionally, most of them are require micromanipulation to control the geometry of the laser, which makes it difficult to control the laser output wavelength and mode. In 2013, Gao et al. (2013) used focused ion beam etching to cut a 9 μm-long GaN nanowire into two nanowires with different lengths at a ratio of 4:3, as shown in Figure 2B. The two nanowires were longitudinally coupled by end-face to form a cleavage coupling cavity, and a single longitudinal mode laser output in the ultraviolet band is obtained. The focus ion beam etching technology can also adjust the cavity length and space, and the precise control of geometric parameters is very beneficial to the manufacturing process. In addition to the physical cutting method, in 2018, Ren et al. (2018) successfully grew six GaAsSb superlattice structures on the same nanowire by controlling the reaction time and injecting materials, and successfully achieved a single-mode output of the near-infrared wavelength using the mode filtering of the cavities of different materials in the nanowires. Organic single-mode nanowire lasers based on longitudinally coupled cavities have also been realized in recent years (Zhang et al., 2017).

The above-mentioned coupling cavities are mostly FP cavities. In 2019, Zhuge et al. (2019a) constructed an nanowire laser based on a FP-WGM-coupled cavity by vertically contacting a CdSSe nanowire and a CdS nanowire. The FP-WGM cavity laser has a threshold value which is about 50% lower than that of the original FP cavity laser. Besides, through a three-dimensional PZT platform to precisely control the coupling point and pump position, the laser achieves reversible tuning of the 42 nm wavelength. This structure is also beneficial for single mode selection in the nanowire laser. By using an FP-WGM cavity by cross-coupling of two CdS nanowires as shown in Figure 2D, we observed one longitudinal mode centered at 517.03 nm with a full width at half maximum (FWHM) of 0.08 nm, which is identical to the previous main peak of wavelength 516.98 nm. Furthermore, the FWHM was decreased from 0.11 to 0.08 nm. These results collectively proved that FP-WGM hybrid cavity coupling successfully strengthens the Q-factors and enables one specific longitudinal mode to lase due to the Vernier effect. By further optimizing the nanowire synthesis process and geometric parameters, a wider range of wavelengths of single-mode lasers with internal reversible tuning are also possible.

## Conclusion

This paper reviews the recent progress of single-mode semiconductor nanowire lasers. In the past decades, a variety of techniques have been proposed for realizing single-mode output laser but these techniques face many problems and challenges. At present, limited by processing technology and other issues, it is difficult to directly process microstructures on nanowires. Besides, the single-mode nanowire lasers introduced in this review are all based on optical pumping, but for scientific research and industrial scenarios, lasers based on electrical pumping have greater application value. Evanescent wave coupled cavities based on loop mirrors and X-shaped cavities face certain difficulties in realizing electrical pumping due to the complexity of the cavity structure. For now, there is still much room for improvement in single-mode semiconductor nanowire lasers, including further reducing the laser pumping threshold, realizing the on-chip integration of single nanowire lasers, and realizing electrically pumped single-mode semiconductor nanowire lasers, etc. With the continuous improvement of preparation technology and integration methods, it is believed that this type of laser will play a more important role in the fields of on chip-communications, optical sensing, displays, and spectroscopy in future.

## Author Contributions

SU and YM drafted the manuscript. YM and QY supervised the project and polished the manuscript. All author contributed to the article and approved the submitted version.

## Conflict of Interest

All authors declare that the research was conducted in the absence of any commercial or financial relationships that could be constructed as a potential conflict of interest.
